# Congenital diaphragmatic hernia and cleft lip and palate: looking for a common genetic etiology

**DOI:** 10.1007/s00383-024-05843-5

**Published:** 2024-10-01

**Authors:** Petra Nord, Ashley H. Ebanks, Petra Peterson, Erik Iwarsson, Matthew T. Harting, Carmen Mesas Burgos, For the Congenital Diaphragmatic Hernia Study Group

**Affiliations:** 1 Department of Pediatric Surgery, Karolinska University Hospital and Karolinska Institutet, Stockholm, Sweden; 2 Department of Plastic and Reconstructive Surgery, Karolinska University Hospital and Karolinska Institutet, Stockholm, Sweden; 3 Department of Pediatric Surgery, McGovern Medical School at UT Health and Children’s Memorial Hermann Hospital, Houston, TX USA; 4 The Fetal Center, Children’s Memorial Hermann Hospital, University of Texas Health Science Center, Houston, TX USA; 5 Department of Molecular Medicine and Surgery, Karolinska Institutet, Stockholm, Sweden; 6 Department of Clinical Genetics, Karolinska University Hospital, Stockholm, Sweden

**Keywords:** Congenital diaphragmatic hernia, Cleft lip and palate, CDH study group, Genetics, Prognosis, Outcome

## Abstract

**Purpose:**

Congenital diaphragmatic hernia (CDH) and cleft lip and/or palate (CL/P) are inborn closure defects. Genetic factors in and outcomes for patients with both anomalies (CDH+CL/P) remain unclear. We aimed to investigate associated genetic aberrations, prevalence of, and outcomes for, CDH+CL/P.

**Methods:**

Data from Congenital Diaphragmatic Hernia Study Group (CDHSG) registry were collected. CL/P prevalence in CDH patients was determined. Genetic abnormalities and additional malformations in CDH+CL/P were explored. Patient characteristics and outcomes were compared between CDH+CL/P and isolated CDH (CDH−) using Fisher’s Exact Test for categorical, and *t*-test or Mann–Whitney *U*-test for continuous, data. *p* < 0.05 was considered statistically significant.

**Results:**

Genetic anomalies in CDH+CL/P included trisomy 13, 8p23.1 deletion, and Wolf-Hirschhorn syndrome (4p16.3 deletion). CL/P prevalence in CDH was 0.7%. CDH+CL/P had lower survival rates than CDH−, a nearly fourfold risk of death within 7 days, were less supported with extracorporeal life support (ECLS), had higher non-repair rates, and survivors had longer length of hospital stay.

**Conclusion:**

Genetic anomalies, e.g. trisomy 13, 8p23.1 deletion, and Wolf-Hirschhorn syndrome, are seen in patients with the combination of CDH and orofacial clefts. CL/P in CDH patients is rare and associated with poorer outcomes compared to CDH−, influenced by goals of care decision-making.

**Supplementary Information:**

The online version contains supplementary material available at 10.1007/s00383-024-05843-5.

## Introduction

Congenital diaphragmatic hernia (CDH) and cleft lip and/or cleft palate (CL/P) are inborn closure defects. The etiology is incompletely understood for the two conditions, but multifactorial models involving genetic as well as environmental factors have been proposed for both malformations [1–3]. CDH and orofacial clefts (OFCs) both occur during the first trimester of the pregnancy and are found in syndromic and non-syndromic variants [4, 5].

CDH affects 2–3 per 10,000 live births [6–8]. The diaphragmatic hernia can be classified by its anatomic location, and the severity of the defect by the CDH Study Group (CDHSG) Staging System for postnatal diaphragm defect size [9]. The heterogenicity of the condition leads to a wide discrepancy in outcomes, with many healthy survivors but also a high morbidity and significant mortality of about 30–50% [5, 8, 10]. More than 50 genetic causes have been associated with CDH [5, 11] and the prevalence of genetic defects in patients with CDH is estimated to approximately 10%, but reported numbers range up to 34% [12–17]. CDH in combination with genetic anomalies has been shown to be associated with poorer outcomes and higher neonatal mortality [18]. However, little is known about the association with craniofacial abnormalities, such as OFCs.

Cleft lip and palate (CLP) is a common birth defect with an incidence of 1–2 per 1000 live births [19, 20]. Orofacial clefts are associated with over 600 identified syndromes, the most common being van der Woude syndrome (VWS), 22q11.2 deletion syndrome, Kallmann syndrome, and Pierre Robin Sequence (PRS) [21]. A few genes are strongly associated with the risk of developing a syndrome which includes an OFC, and several genes have been proposed as candidate genes for increasing the risk of developing non-syndromic OFCs [22]. The severity of the cleft has been seen to increase with the prevalence of a syndrome or an associated malformation [21]. However, previous research on the association with syndromes with other closure defects, such as CDH, is sparse.

Since these two anomalies occur during the same period of intrauterine life, there is a possibility for common contributing genetic or environmental factors, but it is not known which genetic factors contribute to the co-occurrence of these two anomalies. It has not been investigated how the outcomes differ for patients with CDH+CL/P compared to CDH− patients.

### Aim

The aim of this study was to investigate the co-occurrence of a genetic aberration and both malformations, to determine the prevalence of CL/P in patients with CDH, and to describe outcomes for these patients compared to patients with isolated CDH.

## Methods

### Data sources

In this registry-based, international cohort study, data were retrieved from the central registry of the CDHSG. The CDHSG registry is the largest database on CDH and involves patients reported from 133 centers providing care for CDH patients (specified in Appendix 1), with 83 centers active as per the data retrieval for this study. The database collects prospective data, until death or discharge, for all CDH patients born at or transferred to the participating centers, including infants who die in the delivery room or survive only a few hours in the participating centers. The form for registration of patients to the CDHSG in the current version (version 5) can be seen in Appendix 2. The CDHSG registry has been approved for use by the Institutional Review Board of the McGovern Medical School at UT Health in Houston (HSC-MS-03-223).

### Study design and study population

**Fig. 1:**
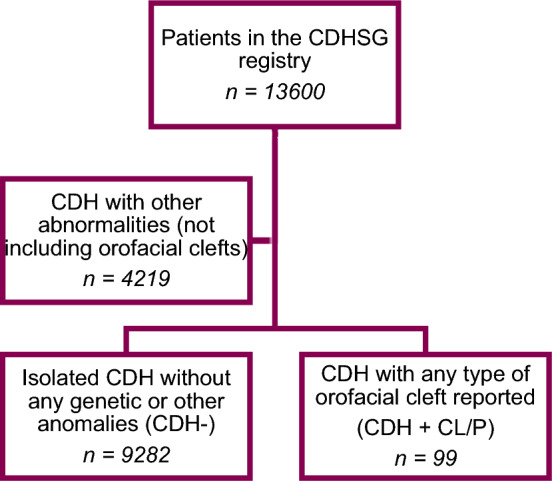
Flowchart of the cases included from the CDHSG registry. Exclusion and subgrouping of patients from the CDHSG registry into two cohorts, CDH+CL/P and CDH−. *CDH* congenital diaphragmatic hernia, *CDHSG* Congenital Diaphragmatic Hernia Study Group, *CL/P* cleft lip and/or cleft palate

All infants reported to the registry of the CDHSG between its inception in 1995 through August 2023 were included in the study. Data from patients entered in the registry with CDH and an associated orofacial cleft (CDH+CL/P) was analyzed and compared to data from patients with isolated CDH (CDH−). CDH+CL/P was defined as all patients entered in the registry with cleft lip with or without cleft palate (CL±P), or isolated cleft palate (ICP), or orofacial cleft registered as an associated anomaly. Isolated CDH was defined as patients with CDH but without any registered genetic, cardiac, or other anomaly (Fig. 1).

### Variables

The CDH+CL/P group was examined with respect to all genetic aberrations recorded in the cohort and to additional reported malformations. The methods used for chromosome analysis during the study period were not registered for CDH+CL/P patients, but the long study period implies that both karyotype, microarray, and, lately, whole exome sequencing/whole genome sequencing have been used.

Subgroup analysis was performed on CDH+CL/P and CDH−. The overall prevalence of CDH+CL/P in the registry was determined. The groups CDH+CL/P and CDH− were analyzed and compared with respect to survival rate, time to death, risk of death within and after the first 7 days of life (DOL), survival rate post surgical repair, defect side (right, left, or bilateral), defect size according to the CDHSG Staging System, use of extracorporeal life support (ECLS), timing and type of surgical repair (of the diaphragmatic hernia), rate of non-repairs, length of hospital stay (LOS), need for oxygen at 30 DOL, and persistent pulmonary hypertension of the newborn (PPHN). The groups were also compared with respect to descriptive parameters, i.e. birth weight, estimated gestational age (EGA), observed/expected lung-to-head ratio (O/E LHR), sex, and rate of prenatal CDH diagnosis.

### Statistics

The analyzed data was presented in absolute values (*n*), percentages (%), odds ratio (OR), hazard ratio (HR) and 95% confidence intervals (CI) for categorical variables. For continuous variables with a presumed normal distribution, data was presented in mean values and standard deviations (SD). Continuous variables with a skewed distribution were presented in medians and interquartile ranges (IQR). For categorical data, Fisher’s Exact test was performed to investigate differences between the groups. For continuous variables with a normal distribution, *t*-test was used, and for continuous variables with a skewed distribution, Mann–Whitney *U*-test was used. Logistic and Cox PH regression was used when appropriate to quantify the strengths of possible associations. Cumulative incidence curves for competing risks were used to illustrate the probabilities of death versus operation and of discharge versus no discharge. The Kaplan Meier survival analysis was used to illustrate time to death after repair and time to death after surviving to 7 DOL. Statistical significance was defined as *p* < 0.05. Analyses were performed using SPSS® version 24.

## Results

Out of the 13,600 patients in the CDHSG registry, 99 patients (0.7%) had a reported orofacial cleft of some sort. The baseline characteristics for the groups CDH+CL/P and CDH− are shown in Table 1. The patients in the CDH+CL/P group had a significantly lower birth weight and a significantly lower EGA than patients with isolated CDH. In the CDH+CL/P group, 76 patients (76.8%) had additional abnormalities reported. The single most common malformation was atrial septal defect (ASD), which was reported in 34 patients. Other commonly reported abnormalities were polydactyly (8 patients) and ventricular septal defect (VSD) (6 patients).

**Table 1:** Patient characteristics

	CDH+CL/P (*n* = 99)	CDH− (*n* = 9282)	*p*-value	OR (95% CI)
Birth weight (kg), mean ± S.D	2.55 ± 0.75	3.02 ± 0.60	<*0.001*	
EGA (weeks), mean ± S.D	36.49 ± 2.83	37.73 ± 2.22	<*0.001*	
O/E LHR (%), mean ± S.D	33.61 ± 23.87	42.81 ± 18.78	0.195	
Male, % (*n*)	61.6 (61)	58.7 (5435)	0.316	1.13 (0.75–1.70)
Prenatal diagnosis, % (*n*)	61.6 (61)	65.8 (6085)	0.222	0.84 (0.56–1.26)

A descriptive comparison of the groups CDH+CL/P and CDH−

The *p*-values considered statistically significant shown in italics

*CDH*− isolated congenital diaphragmatic hernia, *CDH+CL/P* congenital diaphragmatic hernia and any orofacial cleft, *CI* confidence interval, *EGA* estimated gestational age, *O/E LHR* observed/expected lung-to-head ratio, *OR* odds ratio, *S.D.* standard deviation

Twenty-two (22.2%) of the 99 patients with CDH+CL/P had reported genetic anomalies. Genetic abnormalities were most common in the CDH+CL+P group (24.4%) and least common in CDH+ICP patients (18.2%). Genetic aberrations reported more than once in the cohort were trisomy 13 (reported in four patients, out of which three had CL+P and one had ICP), 8p23.1 deletion (reported in five patients, all with ICP), and Wolf-Hirschhorn syndrome/4p16.3 deletion (reported in two patients, both with CL-P). Only seven (31.8%) of the patients with reported genetic anomalies survived to discharge.

**Fig. 2:**
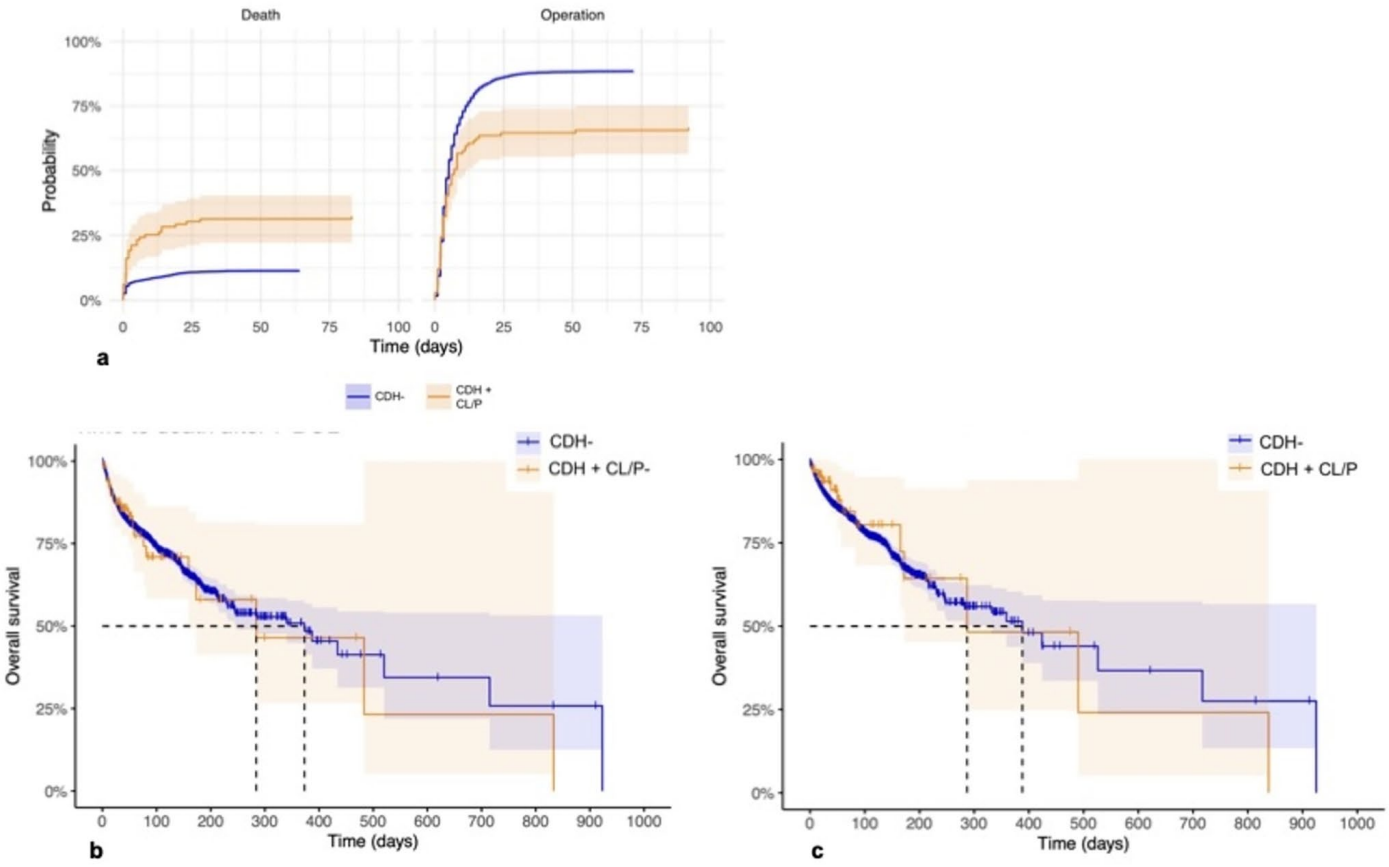
**a**–**c** Death and surgical repair. *Orange line* CDH+CL/P. *Blue line* CDH−. **a** Cumulative incidence curves for competing risk, illustrating the probability of operation or death over time, measured as days since birth. **b** Time to death after surviving to 7 DOL. **c** Time to death after surgical repair. *CDH*− isolated congenital diaphragmatic hernia, *CDH+CL/P* congenital diaphragmatic hernia and any orofacial cleft, *DOL* days of life

The two cohorts were compared with respect to outcomes with the results summarized in Table 2. Survival rates for the patients in the CDH+CL/P group were significantly lower compared to the CDH− group (54.5 vs. 77.7%, *p* < 0.001), with a 3.9 times higher risk of death within 7 days (*p* < 0.001). The probabilities of dying or undergoing surgery of the CDH for the two groups at different points in time can be visualized in Fig. 2a. However, there was no significant difference in risk of death if the patient had survived the first 7 DOL (HR 1.07, CI 1.21–4.05, *p* = 0.8). Kaplan–Meier estimates of time to death after surviving to 7 DOL are shown in Fig. 2b. The rates of patch repair, as a proxy of defect severity, were similar; however, the rates of non-repair were significantly higher in the CDH+CL/P group (32.3%) compared to in the CDH− group (11.3%, *p* < 0.001). The difference in risk of surgical repair was found to be statistically significant only after 8 DOL (HR 0.21, CI 0.12–0.61, *p* = 0.002). Survival curves for deceased patients after surgical repair are visualized in Fig. 2c.

**Table 2:** Patient outcomes

	CDH+CL/P (*n* = 99)	CDH− (*n* = 9282)	*p*-value	OR (95% CI)
Survival, % (*n*)	54.5 (54)	77.7 (7212)	<*0.001*	0.34 (0.23–0.51)
Death within 7 DOL, % (*n*)	26.3 (26)	8.4 (784)	<*0.001*	3.86 (2.45–6.08)
Survival post repair, % (*n*)	80.6 (54)	87.5 (7198)	0.071	0.59 (0.32–1.09)
Defect side, % (*n*)			<*0.001*	
Left	76.8 (76)	84.3 (7817)		
Right	18.2 (18)	15.2 (1411)		
Bilateral	5.1 (5)	0.5 (44)		
Defect size, % (*n*)			0.628	
A	16.7 (2)	14.8 (822)		
B	33.3 (4)	40.4 (2251)		
C	25.0 (3)	32.6 (1819)		
D	25.0 (3)	12.2 (680)		
ECLS, % (*n*)	15.2 (15)	29.3 (2713)	<*0.001*	0.43 (0.25–0.75)
Repair type, % (*n*)			1.000	
Patch	50.0 (33)	50.3 (4112)		
Primary	50.0 (33)	49.5 (4050)		
Overlay	0.0 (0)	0.2 (17)		
Non-repair, % (*n*)	32.3 (32)	11.3 (1053)	<*0.001*	0.27 (0.18–0.41)
O_2_ at 30 DOL, % (*n*)	50.8 (32)	43.3 (3099)	0.143	0.74 (0.45–1.21)
Treatment PPHN, % (*n*)	70.6 (12)	63.2 (4161)	0.361	1.40 (0.49–3.97)
Time to death (DOL), median (IQR)	5 (1–34.5)	14 (2–28)	0.308	
Time to repair (DOL), median (IQR)	4 (2–8)	4 (2–8)	0.133	
Time to discharge (DOL), median (IQR)	49 (26–104.5)	35 (21–61)	*0.003*	

Outcomes for and analyses of the differences between the groups CDH+CL/P and CDH−

The *p*-values considered statistically significant shown in italics

*CDH*− isolated congenital diaphragmatic hernia, *CDH+CL/P* congenital diaphragmatic hernia and any orofacial cleft, *CI* confidence interval, *DOL* days of life, *ECLS* extracorporeal life support, *IQR* interquartile range, *OR* odds ratio, *PPHN* persistent pulmonary hypertension of the newborn, *S.D.* standard deviations

**Fig. 3:**
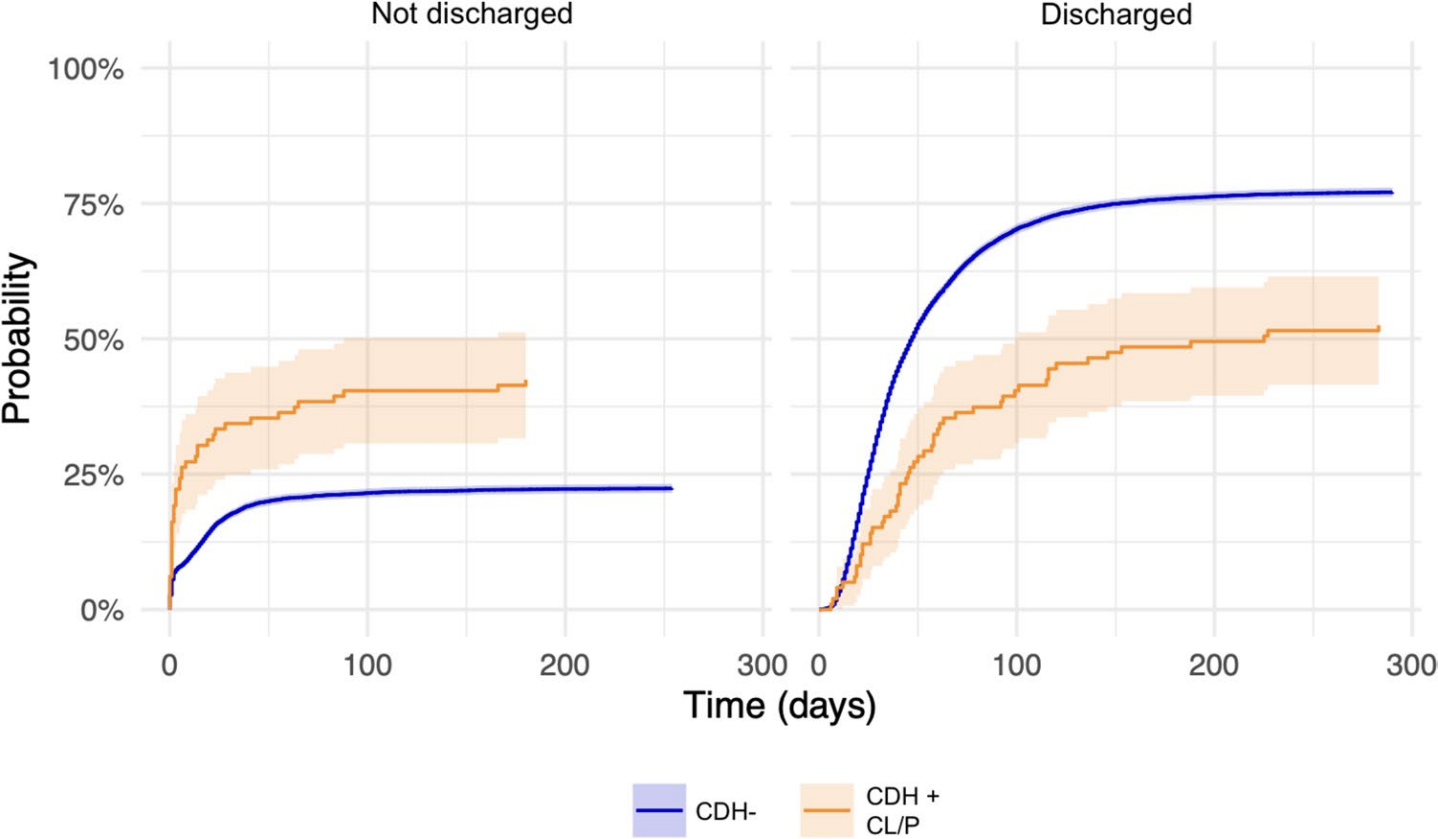
Hospital discharge. Cumulative incidence curves for competing risks, illustrating the probability of being discharged or not over time, measured as days since birth. *CDH*− isolated congenital diaphragmatic hernia, *CDH+CL/P* congenital diaphragmatic hernia and any orofacial cleft

Right sided and bilateral CDH was more common in patients with an associated orofacial cleft (18.2 and 5.1%, respectively), compared to isolated CDH (15.2 and 0.5%, respectively, *p* < 0.001). Patients with CDH+CL/P were also supported with ECLS less often (15.2 vs. 29.3%, *p* < 0.001). The need for oxygen at 30 DOL was higher and PPHN requiring treatment was more often seen in CDH+CL/P, although not statistically significant. The median LOS was longer for the CDH+CL/P group (49 DOL) compared to CDH− (35 DOL, *p* = 0.003). The probabilities of a patient in either group being discharged or not can be visualized in Fig. 3. Furthermore, patients in the CDH+CL/P group were half as likely as CDH− patients to be discharged at all (HR 0.49, CI 0.37–0.63, *p* < 0.001).

## Discussion

Whether there is an association between CDH and CL/P and what the outcomes for these patients are have not previously been investigated, neither are the genetic and environmental factors contributing to the two malformations fully known. Since these congenital closure defects arise during the same period of intrauterine life, there could be common genetic factors influencing the development of both abnormalities. Trisomy 13, deletion of 8p23.1, and Wolf-Hirschhorn syndrome/4p16.3 deletion were recorded multiple times in CDH+CL/P patients. Based on the registered data, the prevalence of OFC in patients with CDH was estimated to 0.7%, which is markedly higher than the 1–2 per 1000 live births that is reported to be the overall prevalence of CL/P [19]. Hence, it seems that OFCs are somewhat more common in infants with CDH compared to in the non-CDH population. That the CDH+CL/P cohort is generally sicker was shown by higher odds of death early after birth, higher rates of non-repair, and a lower chance of and longer time to discharge. However, after surviving to 7 DOL and after surgical repair, survival rates were not significantly different between groups. Even though the results were not significant, probably due to a low number of CDH+CL/P patients with O/E LHR reported, the severity of lung hypoplasia was shown to be more pronounced in this group, shown by lower O/E LHR values.

Multiple studies have pointed towards a heterogenic etiology for CDH with varying degrees of genetic determinants [10, 23]. This study found 22.2% of the CDH+CL/P patients had registered genetic anomalies, compared to the approximately 10% in all CDH patients that earlier studies have reported, although reports range up to 34% [12–17]. This relatively large number points to a higher rate of genetic abnormalities in CDH+CL/P patients. However, it is not the highest number reported, with one possible explanation being that CDH+CL/P patients have another genetic background, to a smaller degree associated to chromosomal abnormalities. That the diagnostic methods have evolved during the study period can also influence the comparison with other studies. Moreover, we cannot exclude the possibility that not all patients entered in the registry have undergone chromosome analysis.

This is the first study to investigate which genetic aberrations occur in patients with both CDH and OFC. Both the deletions (deletion of 8p23.1 and deletion of 4p16.3) and trisomy 13 described in this study have already been reported with CDH, and with CL/P, although to different extents. Trisomy 13 is a chromosomal abnormality with a strong association to CLP [24, 25], and also one of the more common aneuploidies described together with CDH [24], with high antenatal mortality and a survival for live births of less than 10% [26]. Due to the already established associations, the finding is not surprising.

The deletions identified in this study both involve intervals known to be associated with CDH and CL/P. The 8p23.1 deletion is known as a CDH “hot spot”, with an incomplete penetrance of about 50% [27]. There are also reports of the deletion, and the duplication, of 8p23.1 occurring together with an OFC [28, 29]. A gene in this chromosome segment hypothesized to contribute to the development of CDH is GATA-binding protein 4 (GATA4) [10], a transcription factor associated with retinoic acid signaling which is expressed in the developing heart and diaphragm [30]. On the other hand, the 4p16.3 deletion causes Wolf-Hirschhorn syndrome (WHS, MIM: 194190), which is characterized by growth and mental retardation, cardiac anomalies, and dysmorphic craniofacial features [23, 31]. WHS includes an OFC in about 25% of cases [32, 33]. Although not a common feature of the syndrome, CDH has been reported in more than a dozen WHS patients, with multiple genes identified whose haploinsufficiency could contribute to CDH [10, 23, 34]. In summary, the chromosomal abnormalities found in this study correspond well with previous literature on the hypothesized genetic backgrounds of CDH and CL/P.

As far as the authors are aware, the outcomes for patients with CDH and an associated OFC have not been investigated earlier. Although not surprising that outcomes are worse than for patients with isolated CDH, it is noticeable that the OR for death within 7 DOL is almost four times higher for the CDH+CL/P cohort, and that these patients are half as likely to eventually be discharged. The results suggest that, if not operated on at an early age, CDH+CL/P patients have a lower chance of undergoing surgery at all. The significantly higher rates of non-repair and lower rates of ECLS point towards different tendencies in the care given to and resources put into these patients, indicating a sicker and more fragile group. One could speculate that the reduced rate of ECLS indicates a group with a less severe condition but, however, the similarities in defect size and PPHN requiring treatment (as a proxy of severity of the condition) point towards that, presumably, both groups would be needing ECLS to a similar extent. It is therefore plausible that these lower numbers indicate that the infants are presumed beforehand to be too sick to benefit from receiving ECLS.

### Strengths and limitations

The main strength of this study is the broad extent of data. Being both multinational and multi-center, the CDHSG registry includes patients from a wide geographical spectrum. We focused on all infants ever to have been reported to the registry, which provides for the largest material possible from the registry. However, the study also has its limitations, mainly due to reporting of data being both limited and varying over time. Some of the parameters, e.g. hernia size, PPHN, and O/E LHR, were introduced in recent versions of the CDHSG reporting form, which means data is missing for a large share of the CDH+CL/P patients. This could decrease the chance of significant results and potentially skew the results, since some studies have found that outcomes have improved for CDH patients over time [8, 35]. As the CDHSG registry does not specify whether associated abnormalities are not present, not detected, or not registered, there is a level of uncertainty regarding this parameter. The methods, protocols, and availability for genetic testing has also changed a lot during the period that the data were collected. Which genetic tests have been performed, if any at all, is not recorded in the current data set, also limiting the interpretation of the results regarding possible genetic factors. As the data collection only comprises the time of in-hospital stay, it should also be noted that it does not consider any events occurring after patients are discharged from the centers.

### Clinical applications

This study shines a light on the outcomes for infants with a rare combination of congenital malformations, a combination which has not been researched before. Research on infants with CDH influences guidelines for clinical decision making by pediatricians involved in these patients within both surgery and medicine. Research on infants with CDH is also of importance to the families of patients with CDH as it can help improve prenatal counselling and risk prediction [36]. In this context it is also relevant how the co-occurrence of the two malformations CDH and CL/P affects the expected outcomes. Although not overwhelming, nor previously completely undiscovered, the reported genetic anomalies found in the CDH+CL/P group could be of clinical relevance for parents with children with these genetic anomalies. The next step is to perform comprehensive genetic analysis in these cases, using whole genome sequencing in trio format. This will facilitate the study of monogenic causes in the CDH+CL/P group. With a larger part of the genetic factors elucidated, genetic testing and counseling could influence the advising and the clinical management in the context of prenatally diagnosed CDH, to better help families and individuals with CDH, with or without CL/P.

## Conclusions

Genetic aberrations such as deletion of 8p23.1, Wolf-Hirschhorn syndrome, and trisomy 13 are associated with the co-occurrence of CDH and OFC. CL/P in patients with CDH is rare, with a reported prevalence of 0.7%. Compared to cases with isolated CDH, these patients have lower survival rates and higher odds of dying within 7 DOL, require longer hospital stays, are less often offered ECLS, and have higher rates of non-repair. However, if surviving the first week of life and if undergoing surgical repair, the survival of CDH+CL/P patients does not seem to be significantly lower than that of children with CDH−. Decision-making regarding goals of care for these patients seems to influence the outcome.

## Supplementary information

Below is the link to the electronic supplementary material.

Supplementary file1 (DOCX 27 KB)

Supplementary file2 (DOCX 101 KB)

## Data Availability

The data that support the findings of this study are not openly available due to reasons of sensitivity and are available from the corresponding author upon reasonable request. The data were retrieved upon request from the Congenital Diaphragmatic Hernia Study Group, Department of Pediatric Surgery at University of Texas Health Science Center at Houston. https://med.uth.edu/pediatricsurgery/research/research-centers-and-programs/cdhsg/.
