# Supplementary material for: Congenital diaphragmatic hernia and cleft lip and palate: looking for a common genetic etiology
Source: Pediatr Surg Int. 2024 Oct 1;40(1):259. doi: 10.1007/s00383-024-05843-5 (PMC11445286; doi:10.1007/s00383-024-05843-5)
Supplement: Supplementary file 1 — Supplementary file1 (DOCX 27 KB) [file 383_2024_5843_MOESM1_ESM.docx]

**Appendix 1**. Centers used for the CDHSG registry in alphabetical order.

| Hospital | City | State/  Province | Country |
| --- | --- | --- | --- |
| Advocate Lutheran General Hospital | Park Ridge | IL |  |
| Akron Children's Hospital | Akron | OH |  |
| Alberta Children's Hospital | Calgary | AB | Canada |
| Ann & Robert H. Lurie Children's Hospital of Chicago | Chicago | IL |  |
| Arkansas Children's Hospital | Little Rock | AR |  |
| Astrid Lindgren Children's Hospital | Stockholm |  | Sweden |
| Azienda Ospedaliera Papa Giovanni XXIII | Bergamo |  | Italy |
| BC Children's & Women's Health Centre | Vancouver | BC | Canada |
| Boston Children's Hospital | Boston | MA |  |
| Cairo University Pediatric Hospital (Aboul Reesh) | Cairo |  | Egypt |
| Carolinas Medical Center, Levine Children's Hospital | Charlotte | NC |  |
| Cedars Sinai Medical Center | Los Angeles | CA |  |
| Central Hospital Aichi Prefectural Colony | Kasugai | Aichi | Japan |
| Chiba University Hosptal | Chiba-shi | Chiba | Japan |
| Children's Healthcare of Atlanta-Egleston | Atlanta | GA |  |
| Children's Hospital & Research Center Oakland | Oakland | CA |  |
| Children's Hospital at Saint Francis | Tulsa | OK |  |
| Children's Hospital of Buffalo | Buffalo | NY |  |
| Children's Hospital of Georgia - AU Health | Augusta | GA |  |
| Children's Hospital of Illinois at OSF St. Francis Med Center | Peoria | IL |  |
| Children's Hospital of Los Angeles | Los Angeles | CA |  |
| Children's Hospital of Michigan | Detroit | MI |  |
| Children's Hospital of Philadelphia | Philadelphia | PA |  |
| Children's Hospital of San Antonio | San Antonio | TX |  |
| Children's Hospital of Wisconsin | Milwaukee | WI |  |
| Children's Hospital Omaha | Omaha | NE |  |
| Children's Hospitals and Clinics (Minneapolis) | Minneapolis | MN |  |
| Children's Medical Center-Dallas | Dallas | TX |  |
| Children's Memorial Hermann Hospital | Houston | TX |  |
| Children's of Alabama | Birmingham | AL |  |
| Children’s Hospital Colorado | Aurora | CO |  |
| Childrens Hospital at Skanes University Hospital | Lund |  | Sweden |
| Childrens Hospital, University Bonn | Bonn |  | Germany |
| Childrens' Hospital of Orange County | Orange | CA |  |
| Cincinnati Children's Hospital Medical Center | Cincinnati | OH |  |
| Cleveland Clinic Foundation- Children's Hospital | Cleveland | OH |  |
| Connecticut Children's Medical Center | Hartford | CT |  |
| Dayton Chldren's Hospital | Dayton | OH |  |
| Dell Children’s Medical Center of Central Texas | Austin | TX |  |
| Department of Women's and Children's Health, University Hospital of Padua (Italy) | Padua |  | Italy |
| Duke Children's Hospital & Health Center | Durham | NC |  |
| Far Eastern Federal University, School of Medicine | Vladivostok |  | Russia |
| Freie Universitat Berlin | Berlin |  | Germany |
| Golisano Children’s Hospital at Strong | Rochester | NY |  |
| Hasbro Children's Hospital, Brown Medical School | Providence | RI |  |
| Helen DeVos Children's Hospital | Grand Rapids | MI |  |
| Hershey Medical Center | Hershey | PA |  |
| Hospital Clinico Universidad Católica de Chile | Santiago | RM | Chile |
| HSC-Children's Hospital Winnipeg and University of Manitoba | Winnipeg | MB | Canada |
| IRCCS Fondazione Ca' Granda Ospedale Maggiore Policlinico | Milano |  | Italy |
| James Whitcomb Riley Children's Hospital | Indianapolis | IN |  |
| Johns Hopkins All Children’s Hospital | St Petersburg | FL |  |
| Johns Hopkins Children's Center | Baltimore | MD |  |
| Juan P. Garrahan Children Hospital | Buenos Aires |  | Argentina |
| Juntendo University Hospital | Tokyo |  | Japan |
| Juntendo University Urayasu Hospital | Urayasu-shi | Chiba | Japan |
| Kanagawa Children's Medical Center | Yokohama-shi | Kanagawa | Japan |
| Kindai University Hospital | Osakasayama-shi | Osaka | Japan |
| Kobe Children's Hospital | Kobe-shi | Hyogo | Japan |
| Kyoto Prefectural University of Medicine | Kyoto |  | Japan |
| Kyusyu University Hospital | Fukuoka-shi | Fukuoka | Japan |
| La Paz University Hospital | Madrid |  | Spain |
| Le Bonheur Children’s Medical Center | Memphis | TN |  |
| Loma Linda University Children's Hospital | Loma Linda | CA |  |
| Lucile Salter Packard Children's Hospital | Palo Alto | CA |  |
| Massachusetts General Hospital | Boston | MA |  |
| Mattel Children's Hospital at UCLA | Los Angeles | CA |  |
| Mayo Clinic | Rochester | MN |  |
| Miami Valley Hospital | Dayton | OH |  |
| Mie University School of Medicine Hospital | Tsu-chi | Mie | Japan |
| Monroe Carell Jr. Children's Hospital at Vanderbilt | Nashville | TN |  |
| MUSC Health Shawn Jenkins Children's Hospital | Charleston | SC |  |
| Nagoya University Hospital | Nagoya-shi | Nagoya | Japan |
| National Center for Child Health and Development | Tokyo |  | Japan |
| Nationwide Children’s Hospital | Columbus | OH |  |
| Norton Children's Hospital | Louisville | KY |  |
| Ochsner Hospital for Children | New Orleans | LA |  |
| Oklahoma Children's Hospital at OU Health | Oklahoma City | OK |  |
| Osaka University Hospital | Suita-shi | Osaka | Japan |
| Osaka Women's and Children's Hospital | Izumi-shi | Osaka | Japan |
| Ospedale Pediatrico Bambino Gesù | Rome |  | Italy |
| Palmetto Health Richland | Columbia | SC |  |
| Phoenix Children's Hospital | Phoenix | AZ |  |
| Polish Mother's Memorial Hospital Research Institute | Lodz |  | Poland |
| Radboud University Nijmegen Medical Centre | Nijmegen |  | The Netherlands |
| Rady Children's Hospital | San Diego | CA |  |
| Rainbow Babies and Children Hospital | Cleveland | OH |  |
| Randall Children's Hospital at Legacy Emanuel | Portland | OR |  |
| Research Center for Obstetrics, Gynecology and Perinatology | Moscow |  | Russia |
| Rockford Memorial Children's Hospital | Rockford | IL |  |
| Royal Alexandra Hospital | Edmonton | Alberta | Canada |
| Royal Children's Hospital | Parkville | Victoria | Australia |
| Royal Hospital for Children, Glasgow | Glasgow |  | Scotland |
| Royal Manchester Children's Hospital | Manchester |  | England |
| Santa Rosa Children's Hospital | San Antonio | TX |  |
| Seattle Children's Hospital | Seattle | WA |  |
| Shands Children's Hospital/University of Florida | Gainesville | FL |  |
| Shizuoka Children's Hospital | Shizuoka-shi | Shizuoka | Japan |
| Sidra Medicine | Doha |  | Qatar |
| Sophia Children's Hospital | Rotterdam |  | The Netherlands |
| SSM Health Cardinal Glennon Children's Hospital/Saint Louis University School of Medicine | St. Louis | MO |  |
| St. Joseph's Hospital and Medical Center | Phoenix | AZ |  |
| St. Louis Children's Hospital | St. Louis | MO |  |
| St. Paul Campus Children's Minneapolis | Minneapolis | MN |  |
| Stollery Children's Hospital | Edmonton | AB | Canada |
| Sydney Children's Hospital | Randwick | NSW | Australia |
| T.C. Thompson Hospital | Chattanooga | TN |  |
| Texas Children's Hospital | Houston | TX |  |
| The Hospital for Sick Children | Toronto | Ontario | Canada |
| The Queen Silvia Children’s Hospital SU/Östra | Gothenburg |  | Sweden |
| Tsukuba University Hospital | Tsukuba-shi | Ibaraki | Japan |
| Tufts Medical Center | Boston | MA |  |
| UNC Children's Hospital | Chapel Hill | NC |  |
| Universitatsklinikum Mannheim gGmbH | Mannheim |  | Germany |
| University Childrens Hospital | Uppsala |  | Sweden |
| University Hospital Gasthuisberg | B-3000 Leuven |  | Belgium |
| University Malaya Medical Centre | Kuala Lumpur |  | Malaysia |
| University of California San Diego | San Diego | CA |  |
| University of Chicago | Chicago | IL |  |
| University of Kentucky Medical Center | Lexington | KY |  |
| University of Michigan, C.S. Mott Children's Hospital | Ann Arbor | MI |  |
| University of Mississippi Medical Center | Jackson | MS |  |
| University of Nebraska Medical Center | Omaha | NE |  |
| University of North Carolina | Chapel Hill | NC |  |
| University of Texas Medical Branch at Galveston | Galveston | TX |  |
| University of Utah and Primary Children's Hospital | Salt Lake City | UT |  |
| University of Wisconsin Hospital and Clinics | Madison | WI |  |
| UPMC Children's Hospital of Pittsburgh | Pittsburgh | PA |  |
| UVA Children's Hospital | Charlottesville | VA |  |
| Wilford Hall USAF Medical Center | Lackland AFB | TX |  |
| Winnie Palmer Hospital for Women & Babies | Orlando | FL |  |
| Wolfson Children's Hospital | Jacksonville | FL |  |
| Yale New Haven Children's Hospital | New Haven | CT |  |
