# Supplementary material for: Congenital diaphragmatic hernia and cleft lip and palate: looking for a common genetic etiology
Source: Pediatr Surg Int. 2024 Oct 1;40(1):259. doi: 10.1007/s00383-024-05843-5 (PMC11445286; doi:10.1007/s00383-024-05843-5)
Supplement: Supplementary file 2 — Supplementary file2 (DOCX 101 KB) [file 383_2024_5843_MOESM2_ESM.docx]

**Appendix 2**. Data form for registration of patients to the CDHSG registry, version 5.

**CONGENITAL DIAPHRAGMATIC HERNIA FORM**

**(To be used for patients born on or after 1/1/2021)**

Center #:       Patient #:

Date of Birth:       Time of Birth:

Inborn

Outborn: Date of Admission:       Time of Admission:

Sex:  M  F

Race:  Asian  Black  Hispanic  Native American  White  Other:

EGA (at birth):       weeks Birthweight:       kg APGARs (1/5):      /

Method of Delivery:  Vaginal (Spontaneous)  Vaginal (Induced)

C-section (Elective)  C-section (Urgent/Non-elective)

Prenatal diagnosis of CDH:  Yes  No

If **Yes**, diagnosis made at       weeks gestation

Prenatal steroids:  Yes  No  Unknown

Surfactant (after delivery):  Yes  No  Unknown

Genetic studies (Check all that apply and please provide DX if known):

Chromosomal analysis (Karyotype):  Sent  Not Sent

Result:  Normal  Abnormal  Pending  Unknown

Chromosomal microarray analysis (CMA):  Sent  Not Sent

Result:  Normal  Abnormal  Pending  Unknown

Sequencing test type:  Not sent

Single gene/panel; Result:  Normal  Abnormal  VUS  Pending  Unknown

Whole Exome (WES); Result:  Normal  Abnormal  VUS  Pending  Unknown

Whole Genome (WGS); Result:  Normal  Abnormal  VUS  Pending  Unknown

Diagnosis made/Comment: please describe:

Other Anomalies-If Yes, please describe:

**Associated Structural Cardiac Anomalies (Check all that apply):**

ASD

VSD

AVSD (AV Canal)

Pulmonic/Pulmonary Valvular/Artery Stenosis/Atresia

TOF (Tetralogy of Fallot)

Coarctation of Aorta  Hypoplastic aortic arch

TOGV (Transposition of Great Vessels or Transposition of Great Arteries)

### Truncus Arteriosus

Complex biventricular anatomy (i.e. heterotaxy syndrome)

Total Anomalous Pulmonary Venous Return (TAPVR)

Single Ventricle Variant (hypoplastic left heart syndrome)

Other- please describe:

Pulmonary Hypertension/Cardiac Dysfunction (CDH-PH/CD):

First ECHO done on Date:       Time:

PH:  None  < 2/3 systemic  between 2/3 and systemic  > systemic  Present but cannot quantify

PDA:  L to R  Bidirectional  R to L  No shunt (closed)

Atrial shunt:  L to R  Bidirectional  R to L  No shunt (closed)

Tricuspid regurgitation peak velocity:       m/sec Systemic BP       /

Septal position:  Normal  Flattened  Further left deviation

RV size:  Normal  Dilated

RV function:  Normal  Impaired

LV size:  Below normal  Normal  Dilated

LV function:  Normal  Impaired

Plasma level       (pg/ml) of pro-BNP; Date:

Second (closest to pre-op) ECHO done on Date:

PH:  None  < 2/3 systemic  between 2/3 and systemic  > systemic  Present but cannot quantify

PDA:  L to R  Bidirectional  R to L  No shunt (closed)

Atrial shunt:  L to R  Bidirectional  R to L  No shunt (closed)

Tricuspid regurgitation peak velocity:       m/sec Systemic BP       /

Septal position:  Normal  Flattened  Further left deviation

RV size:  Normal  Dilated

RV function:  Normal  Impaired

LV size:  Below normal  Normal  Dilated

LV function:  Normal  Impaired

Plasma level       (pg/ml) of pro-BNP; Date:

Last (closest to end of hospital course) ECHO done on Date:

PH:  None  < 2/3 systemic  between 2/3 and systemic  > systemic  Present but cannot quantify

PDA:  L to R  Bidirectional  R to L  No shunt (closed)

Atrial shunt:  L to R  Bidirectional  R to L  No shunt (closed)

Tricuspid regurgitation peak velocity:       m/sec Systemic BP       /

Septal position:  Normal  Flattened  Further left deviation

RV size:  Normal  Dilated

RV function:  Normal  Impaired

LV size:  Below normal  Normal  Dilated

LV function:  Normal  Impaired

Plasma level       (pg/ml) of pro-BNP; Date:

**Treatment of Pulmonary Hypertension (CDH-PH):**

| Check if Used (use “Other” for additional courses of iNO as well as unlisted medications) | | Date Started | Date Ended |
| --- | --- | --- | --- |
|  | Inhaled Nitric Oxide |  |  |
|  | Sildenafil  Oral  iv |  |  |
|  | Endothelial Receptor Blockade |  |  |
|  | Prostacyclin |  |  |
|  | Alprostadil (PGE1) |  |  |
|  | Milrinone |  |  |
|  | Other (specify): |  |  |
|  | Other (specify): |  |  |

**Ventilation:**

Intubated at: Date:       Time:

Extubated at: Date:        Never extubated

Values in the first 24 hours of life (pre-ECLS):

| Highest **pre**-ductal PaO_2_:        mm Hg (or kPascal)  O_2_ sat:       % | Highest **post**-ductal PaO_2_:        mm Hg (or kPascal)  O_2_ sat:       % |
| --- | --- |
| Highest PaCO_2_:        mm Hg (or kPascal) | Lowest PaCO_2_:        mm Hg (or kPascal) |

**ECLS Data:**

Placed on ECLS: started at Date:       Time:

ended at Date:       Time:

ECLS Mode:  VA  VA (+V)  VV (DL)  VV to VA

Last ABG (blood gas) before going on ECLS:

PaO_2_:       mm Hg (or kPascal) Preductal Postductal

O_2_ Sat:       % Preductal Postductal

PaCO_2_:       mm Hg (or kPascal)

Second ECLS run: started at Date:       Time:

ended at Date:       Time:

ECLS Mode:  VA  VA (+V)  VV (DL)  VV to VA

**Side of Diaphragmatic Hernia:**  Left  Right  Bilateral  Central

**Repair:** Done on Date:       Time:        Not repaired

Diaphragm Defect:  A  B  C  D


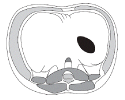

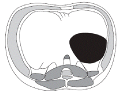

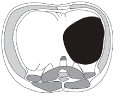

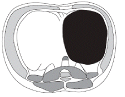


(Have surgeon identify which diagram (A, B, C, D) most closely approximates defect noted intra-operatively. Orientation: diagram is drawn with the diaphragm (defect) on the patient’s left and you are looking up from the abdomen towards the chest)

Type of Diaphragm Repair:  Primary  Patch  Primary repair with Patch overlay

If patch:  PTFE  PTFE/combination patch; combination material:

Muscle flap  Other:

Hernia Sac:  Yes  No

Liver:  Chest  Abdomen

Approach:  Subcostal  Thoracic  Thoracoscopic  Laparoscopic

Both subcostal and thoracic  Other:

Estimated Blood Loss (EBL) at Operation:

**Complications/Other Surgical Procedures** (Check all that apply and provide dates):

|  | Repair of recurrent CDH | Date: |
| --- | --- | --- |
|  | Gastrostomy tube (no fundoplication) | Date: |
|  | Fundoplication, G-tube  Yes  No | Date: |
|  | Lysis of adhesions / surgery for SBO | Date: |
|  | Closure of ventral hernia | Date: |
|  | Bleed requiring reop for hemorrhage control (site:      ) | Date: |
|  | Cardiac catheterization | Date: |
|  | Cardiac surgery (details:      ) | Date: |
|  | Tracheostomy | Date: |
|  | Other surgery (details:      ) | Date: |

**Outcome:**

**Death**: Date of death:       Time of death:

**Survived** to discharge home or transfer

Discharged home or transferred on Date:

Discharged home

Transferred to another hospital

Transferred to in-hospital service for long-term care

Pulmonary Status at **30 Days of Age**:

Extubated and on room air

Extubated and on nasal cannula

Nasal CPAP

Mechanical ventilation

ECMO

Pulmonary status at Time of **discharge/transfer**:

Extubated and on room air

Extubated and on nasal cannula

Nasal CPAP

Mechanical ventilation

Head U/S:  Normal  Abnormal  Not done

Head CT:  Normal  Abnormal  Not done

Cranial MRI:  Normal  Abnormal  Not done

At time of (or closest to) discharge:

Weight:       kg

Feeding at Time of discharge/transfer:

PO (> 50 % feeds po)

NG (≥ 50 % feeds by gavage)

GT (≥ 50 % feeds by G-tube)

Date on full enteral feeds:

GER (Gastro-esophageal reflux) diagnosed:  Yes  No

**Discharge medications** (Check all that apply):

| Respiratory:  Diuretics  Inhaled bronchodilators  Inhaled steroids  iNO  Prostacyclin  Sildenafil  Theophylline  Antibiotics  Seizure medications  Sedatives/analgesics | Gastrointestinal:  Prokinetic agents  Antacids(ranitidine, proton pump inhibitors, etc.)  Erythromycin (used to increase motility)  Hyperalimentation  Cardiac:  Digoxin  Captopril  Aspirin |
| --- | --- |

(Any medications not listed here, please list in comments section below)

Additional Comments about this Patient:

**ULTRASOUND AND MRI DATA COLLECTION: ONLY FOR PRENATALLY DIAGNOSED CDH**

Maternal: Age:       (years) G       P

**FIRST PRENATAL ULTRASOUND:**

Date:

Gestational age at time of exam:       weeks

Side of Hernia:  Left  Right  Bilateral  Central

Sign of Hydrops:  None  Skin edema  Pleural effusion  Pericardial effusion  Ascites

Liver Herniation:

No  Yes- If yes,  <1/3 thoracic cavity height  ≥1/3 thoracic cavity height

LHR:       (range 0.5 – 2.5)

Method used:  Method A  Method B  Method C  Unknown/not stated

Observed/Expected LHR:       (range 0 – 150 %)

Lung/Thorax (L/T) ratio:       (range 0 – 0.5)

Stomach Herniation (Kitano Method- see manual for explanation):
 Grade 0  Grade 1  Grade 2  Grade 3

Stomach Herniation (Cordier Method- see manual for explanation):
Grade 1  Grade 2  Grade 3  Grade 4

Was fetoscopic endoluminal tracheal occlusion (FETO) performed?  No  Yes (If yes, fill in table below)

|  | Balloon IN | Balloon OUT |
| --- | --- | --- |
| Date |  |  |
| EGA |  |  |
| O/E LHR |  |  |
| LHR |  |  |
| O/E TFLV |  |  |
| TFLV |  |  |
| Liver herniation | No  Yes-  If yes,  <1/3 thoracic cavity height  ≥1/3 thoracic cavity height | No  Yes-  If yes,  <1/3 thoracic cavity height  ≥1/3 thoracic cavity height |

Other malformations?  No  Yes - If yes, provide details:

**SECOND PRENATAL ULTRASOUND:**

Date:

Gestational age at time of exam:       weeks

Side of Hernia:  Left  Right  Bilateral  Central

Sign of Hydrops:  None  Skin edema  Pleural effusion  Pericardial effusion  Ascites

Liver Herniation:

No  Yes- If yes,  <1/3 thoracic cavity height  ≥1/3 thoracic cavity height

LHR:       (range 0.5 – 2.5)

Method used:  Method A  Method B  Method C  Unknown/not stated

Observed/Expected LHR:       (range 0 – 150 %)

Lung/Thorax (L/T) ratio:       (range 0 – 0.5)

Stomach Herniation (Kitano Method- see manual for explanation):
 Grade 0  Grade 1  Grade 2  Grade 3

Stomach Herniation (Cordier Method- see manual for explanation):
Grade 1  Grade 2  Grade 3  Grade 4

Other malformations?  No  Yes - If yes, provide details:

**PRENATAL MRI:**

Date:

Gestational age at time of exam:       weeks

Side of Hernia:  Left     Right     Bilateral  Central

Lung volumes:  Left       ml   Right       ml Total (left + right)       ml

TV: Thoracic volume       (ml)

MV: Mediastinal volume       (ml)

Percent Predicted Lung volume (PPLV)       %

PPLV = Total lung volume * 100 / (Thoracic volume – Mediastinal volume)

O/E total lung volume:

Liver: estimated percent of liver in chest:       % (0 = all liver in abdomen / 100 = all liver in chest)

Grading of location of the fetal stomach (Usui Method- see manual for explanation):

Stomach Herniation:    Grade 0       Grade 1       Grade 2       Grade 3

Other malformations?  No     Yes - If yes, provide details:
